# Evaluation of the Veterans Health Administration’s Digital Divide Consult for Tablet Distribution and Telehealth Adoption: Cohort Study

**DOI:** 10.2196/59089

**Published:** 2024-09-09

**Authors:** Jacqueline M Ferguson, James Van Campen, Cindie Slightam, Liberty Greene, Leonie Heyworth, Donna M Zulman

**Affiliations:** 1 Center for Innovation to Implementation Veterans Affairs Palo Alto Healthcare System Menlo Park, CA United States; 2 Division of Primary Care and Population Health Stanford School of Medicine Palo Alto, CA United States; 3 Office of Connected Care/Telehealth Department of Veterans Affairs Central Office Veterans Health Administration Washington, DC United States; 4 Department of Medicine University of California, San Diego School of Medicine San Diego, CA United States

**Keywords:** veterans, health care access, video-based care, telehealth, barriers to care, telemedicine

## Abstract

**Background:**

Video telehealth offers a mechanism to help Veterans Health Administration (VHA) patients overcome health care access barriers; however, many veterans lack a suitable device and sufficient internet connectivity. To address disparities in technology access, VHA established a Connected Device Program that offers veterans loaned video-capable tablets and internet service. In 2020, VHA introduced a national Digital Divide Consult to facilitate and standardize referrals for this resource.

**Objective:**

We sought to evaluate the reach and impact of VHA’s Connected Device Program, leveraging Digital Divide Consult data to determine whether resources are supporting veterans with health care needs and access barriers.

**Methods:**

We examined the reach of VHA’s Connected Device Program using national secondary data from VHA’s electronic health records among 119,926 tablet recipients who received a tablet (April 1, 2020, to February 28, 2023) and 683,219 veterans from the general VHA population. We assessed changes in tablet recipients’ demographic and clinical characteristics before and after implementation of the Digital Divide Consult compared with the general VHA population. We examined the impact of tablets and the consult on adoption of telehealth (ie, video visit use and number of visits) adjusting for differences between tablet recipients and the general VHA population. Finally, we evaluated consult implementation by assessing the use of video-based services by tablet referral reason.

**Results:**

Common reasons for tablet referral included mental health diagnoses (50,367/79,230, 63.9%), distance from a VHA facility >30 miles (17,228/79,230, 21.7%), and social isolation (16,161/79,230, 20.4%). Moreover, 63.0% (49,925/79,230) of individuals who received a tablet after implementation of the Digital Divide Consult had a video visit in the first 6 months of tablet receipt. Some consult reasons were associated with a higher-than-average percentage of video telehealth use, including enrollment in evidence-based mental health programs (74.8% [830/1100] with video use), living >30 miles from a VHA facility (68.3% [10,557/17,228] with video use), and having a mental health diagnosis (68.1% [34,301/50,367] with video use). Tablet recipients had nearly 3 times the likelihood of having a video visit within a month once provided a tablet compared to the general VHA population, with an adjusted risk ratio of 2.95 (95% CI 2.91-2.99) before consult implementation and 2.73 (95% CI 2.70-2.76) after consult implementation. Analyses of telehealth adoption suggested that veterans receiving tablets for mental health care and evidence-based programs have higher rates of video visits, while those who are homebound or receiving tablets for hospice have higher rates of nonuse.

**Conclusions:**

This evaluation of VHA’s Connected Device Program suggests that tablets are facilitating video-based care among veterans with complex needs. Standardization of referrals through the Digital Divide Consult has created opportunities to identify groups of tablet recipients with lower telehealth adoption rates who might benefit from a targeted intervention.

## Introduction

Access to health care is a foundational priority for the US Veterans Health Administration (VHA) [1]. Many veterans experience barriers that impede their use of VHA clinical and social services, including geographic and transportation difficulties, physical and mental health challenges, and socioeconomic stressors [2]. Approximately 2.7 million VHA veterans live in rural or insular areas [3], a scenario that can compound other access barriers.

Video telehealth (ie, delivery of clinical care via video visits) offers a mechanism to overcome health care access barriers. This is particularly the case in a setting, such as VHA, a national health care system in which veterans can receive care from clinicians in remote locations. VHA’s investments in telehealth date back over 20 years [4], a history that facilitated widespread expansion of telehealth during the COVID-19 pandemic [5]. However, video telehealth requires that patients have a suitable device and internet connectivity. By some estimates, 15% of veterans lack home internet [6] and nearly one-third of rural veterans do not access the internet at home [3]. This unequal access to digital technology—or “digital divide”—can translate to disparities in health care access [7]. VHA studies during the COVID-19 pandemic found evidence of a persistent digital divide [8], with a lower likelihood of video visits occurring among VHA patients in rural areas, those who are older, and those with a history of homelessness [9,10].

To enhance access to video telehealth, policymakers and VHA leaders have taken several steps, including removing geographic licensure barriers for providers and establishing partnerships with select major mobile carriers to reduce data fees associated with the use of VHA’s video telehealth platform [8,11]. In 2016, VHA’s Office of Rural Health (ORH) and Office of Connected Care (OCC) launched the Connected Device Program to distribute video-enabled tablets to veterans who had a clinical need that could be met through video telehealth, but who did not have their own device or a suitable data plan [12,13]. To support the ongoing evaluation and improvement of this program, VHA’s Quality Enhancement Research Initiative (QUERI) and ORH funded the Virtual Access QUERI in 2018 [14]. Previous analyses supported by this partnered evaluation have examined tablet distribution patterns; veterans’ experiences with the tablets; and the impact of tablets on mental health care access, continuity, and outcomes [15-19]. One evaluation found that rural veterans are at risk for suicide who received a VHA-issued tablet (compared to those who did not) experienced increases in mental health service use, as well as a 36% reduction in the likelihood of suicide-related emergency department visits [19]. Other assessments by the Virtual Access QUERI have included a survey of tablet recipients to understand their experience with and the remaining barriers to using video telehealth after the provision of a tablet [17,20,21].

The Virtual Access QUERI’s partnership with OCC informed the design and implementation of the Digital Divide Consult, a new referral process for veterans to qualify for devices. Implemented in VHA in September 2020, this new nationwide standardized consult e-referred veterans to VHA social workers to assess the need and eligibility for the VHA-loaned tablet program or for other federal subsidies for a device and connectivity. The standardized consult offered an opportunity to examine the characteristics of tablet recipients and tablet referral patterns in greater detail across the entirety of VHA. As part of the Virtual Access QUERI, we leveraged the implementation of the Digital Divide Consult to conduct an in-depth assessment of the following: (1) tablet recipients’ demographic and clinical characteristics; (2) the monthly use of video care among veterans with a tablet compared to the monthly use among the general VHA population (ie, veterans without a tablet), including changes in use patterns before and after consult implementation; (3) provider-selected reasons for tablet referral according to Digital Divide Consult data; and (4) the use of video-based services by tablet referral reason.

## Methods

### VHA Digital Divide Consult

The Digital Divide Consult is a format-standardized template in VHA’s computerized patient record system (CPRS). Through this consult template, any VHA clinical staff member who manages a veteran’s care can request the provision of a video-enabled device and internet service for the veteran. The provider may also request a peripheral device for use during video appointments (such as a blood pressure monitor or pulse oximeter). After the consult is placed, a social worker reviews the consult, evaluates the veteran’s need for a device and internet service, and places an order to have a device with a data plan shipped directly to the veteran. In addition, the social worker can help the veteran apply for other federal broadband programs [22], including EveryoneOn (discontinued June 1 2024) [23] and Lifeline [24].

Prior to implementation of the Digital Divide Consult (mandatory by September 15, 2020), VHA did not have a nationally standardized process for tablet distribution. While there were explicit criteria for tablets, the national program offices (ORH and OCC) could not reliably track distribution reasons or adherence to the criteria until the implementation of the Digital Divide Consult [13]. Through the Digital Divide Consult, the provider must indicate that the veteran meets one or more criteria for a tablet, which is henceforth referred to as Digital Divide Consult reasons. The Digital Divide Consult expands on the 4 existing consult reasons and includes more detailed device eligibility criteria compared to the original consult (Table 1). In addition, the health care provider placing the consult is required to attest that the veteran will require a video telehealth appointment in the next 90 days.

**Table 1:** Comparison of consult criteria before the Digital Divide Consult.

Pre-Digital Divide Consult criteria	Digital Divide Consult criteria
Transportation issues	No DAV^a^ van or Veterans Transportation Network access Difficulty accessing public transportation No car or access to a ride
Distance or geography	Veteran lives >30 miles from a VHA^b^ medical facility
Homebound or difficulty leaving home	Work, school, or caregiver commitments make in-person visits challenging Health issues make the patient homebound or make in-person visits challenging Any mental health diagnosis Participation in an evidence-based protocol for mental health therapy Veteran has been hospitalized in the past 90 days Social isolation
Other reasons described by the provider	Cost of attending in-person visits is prohibitive Difficulty attending visits at a VHA facility (eg, psychological distress and immunocompromised state) Documented disruptive behavior at a VHA facility Homeless veteran or enrolled in HUD-VASH^c^ The patient does not meet any of the criteria (other reasons described by the provider)

^a^DAV: Disabled American Veterans.

^b^VHA: Veterans Health Administration.

^c^HUD-VASH: US Department of Housing and Urban Development-VA Supportive Housing.

### Veteran Study Sample

Veteran patient data and outpatient visit history were extracted from the VHA Corporate Data Warehouse (CDW), a repository for VHA electronic health records [25]. The evaluation focused on 119,926 tablet recipients who had at least one outpatient VHA visit in 2019. In order to compare tablet recipients to the general VHA population, we also identified a random sample of 683,219 veterans with at least one VHA outpatient visit in 2019, who had not received a tablet. Overall, the study cohort comprised 803,145 veterans.

Tablet recipients were limited to those who received a tablet between April 01, 2020, and February 28, 2023, using the most recent tablet shipment date. This study sample excluded veterans with missing data on their zip code (required for drive time and rurality of residence) or priority enrollment group and those who had less than 1 month of follow-up (ie, tablet recipients who died within 1 month of tablet receipt or veterans from the general VHA population who died in April 2020). Additional details on sample selection and exclusion criteria are available in Multimedia Appendix 1.

### Outcomes

Our outcomes were the occurrence of an outpatient video visit and the number of outpatient video visits in each month between April 1, 2020, and August 31, 2023. As a secondary analysis, we evaluated phone (eg, audio-only) visits. Though video visits are the preferred modality for telehealth at VHA [5], telehealth visits can also be offered via phone owing to patient’s limited broadband access or provider-patient preference [26]. Outpatient video-based and phone-based VHA health care visits were identified using VHA’s Managerial Cost Accounting (MCA) Stop Codes [9]. Veterans were censored in the month of death. We focused posttablet follow-up among tablet recipients to the first 6 months (180 days) of tablet receipt. This timeframe has been used in previous evaluations [15,19,27] because it spans the period of the highest telehealth engagement after tablet receipt and because devices are retrieved on a quarterly basis if they have not been used in the past 90 days.

### Veteran Patient Characteristics

To compare differences between tablet recipients and the general VHA population and to adjust for confounding factors required to ensure exchangeability between the 2 groups, we obtained veteran patient characteristics that have been associated with health care use [9,10,28], using data from a 1-year baseline period (March 11, 2019, to March 10, 2020). The selection of a baseline period in this evaluation is complicated by eligibility criteria (ie, tablet receipt) being met at multiple times as a veteran could be referred for a tablet at any time between April 01, 2020, and February 28, 2023. We selected an unbiased choice of time zero [29] and defined the baseline period prior to the first eligible time (ie, April 01, 2020) and prior to the start of the COVID-19 pandemic.

Characteristics assessed included age, sex, race, ethnicity, marital status, rurality of the home, VHA priority enrollment category, drive time to a primary site of care, number of chronic conditions, and presence of a mental health condition. Race and ethnicity were the most frequent self-identified classifications in patient health records. Missing data on race, ethnicity, or marital status were treated as distinct categories. Veterans’ home addresses were classified into urban, rural, and highly rural designations based on the zip code of their home address [6,10]. We incorporated information from VHA’s priority-based enrollment system, which categorizes veterans into 1 of 8 groups based on their disability rating, income, recent military service, and other factors [6]. We report priority groups in 4 categories: high disability, low or moderate disability, low income, and enrolled without special considerations. Twenty-eight chronic conditions, including 10 mental health conditions, were identified using ICD-10 codes [9,30-33].

To assess the provider-selected criteria for tablet receipt in the Digital Divide Consult, among all veterans (including those without a Digital Divide Consult), we assessed 4 veteran patient characteristics from electronic health care records that have been shown to be associated with differential use of video-based care [10,27]. A history of housing instability and history of homelessness were defined following an adapted methodology from Tsai et al [34] (Multimedia Appendix 1). A history of hospitalization was defined as an inpatient admission in the 180 days before March 1, 2020. A high risk for suicide was derived from the “High Risk for Suicide Patient Record Flag” (HRS-PRF) in patient electronic health care records between March 1, 2019, and March 2020. This patient record flag is used after a suicide risk evaluation or when a patient has had a recent suicide attempt, suicidal preparatory behavior, or suicidal ideation (Multimedia Appendix 1).

We also assessed prior use (eg, during the baseline period) of VHA outpatient care, including prior use of video-based, phone-based, and remote patient monitoring health care (ie, remote blood pressure monitoring) and prior use of primary care, mental health, specialty, and diagnostic or ancillary care [9,10]. See Multimedia Appendix 1 for additional information on the assessment of all veteran patient characteristics.

Digital Divide Consults were identified in the CDW and linked to tablet shipment dates obtained from VHA’s Denver Acquisitions and Logistics Center, which oversees tablet distribution [5,13]. For pre-post analyses of the Digital Divide Consult implementation, we defined the preperiod as April 1, 2020, to October 1, 2020, and the postperiod as October 1, 2020, through the end of follow-up, August 31, 2023. We used October 1, 2020, rather than September 15, 2020 (effective date of consult implementation) to best capture shipped tablets generated from the new consult (ie, to account for the lag between the consult close date and tablet shipment).

### Nationwide Trends in Telehealth and Tablet Distribution in the Study Sample

To describe trends in tablet distribution and nationwide trends in telehealth use (ie, beyond the analytic sample), we tallied all video and phone outpatient care visits nationwide in VHA between January 5, 2019, and September 2, 2023, among primary care, mental health, and subspecialty services. We graphically represented the locally estimated scatter plot smoothed (LOESS) weekly total number of video and phone visits nationwide (ie, line graph) overlaid on a bar graph representing the number of tablets shipped in each week for the 119,926 tablet recipients in the analytic sample.

### Statistical Analysis: Evaluation of VHA’s Connected Device Program and Digital Divide Consult

We performed several analyses to evaluate the VHA’s Connected Device Program and Digital Divide Consult

#### Sociodemographic and Clinical Characteristics of Tablet Recipients and the General VHA Population

We have described the sociodemographic and clinical characteristics of tablet recipients (all and those who received tablets before and after Digital Divide Consult implementation), and compared tablet recipients with the general VHA population.

#### Evaluation of the Digital Divide Consult and Video Telehealth Use

To examine how the Digital Divide Consult influenced video telehealth use while accounting for temporal differences induced by the COVID-19 pandemic, we estimated monthly video visit use as defined by (1) the likelihood of any video visit in a month and (2) the count of video visits in a month. We assigned each person-month between April 01, 2020, and August 31, 2023, to the following categories: category A, person-months without a tablet (includes person-months from the general VHA population and person-months of tablet recipients *before* receiving a tablet); category B, person-months with a tablet among veterans who received their tablet before the implementation of the Digital Divide Consult; and category C, person-months with a tablet among veterans who received their tablet after the implementation of the Digital Divide Consult. Each model had fixed effects for the calendar month, and person-months without a tablet were set as the reference group. Therefore, our models compared the monthly use of video care in any given month between (1) category B divided by category A and (2) category C divided by category A.

By including a fixed effect for the calendar month and by comparing the monthly use of video care in the same month between person-months with a tablet and person-months without a tablet, we accounted for temporal differences in video care use induced by the COVID-19 pandemic. We accounted for differences between tablet recipients and the general VHA population by adjusting for the key patient demographics associated with differential use of video telehealth (ie, age, sex, rurality, etc) and the 4 characteristics similar to those captured in the Digital Divide Consult as described earlier (eg, history of housing instability, history of homelessness, history of hospitalization, and high risk for a suicide patient risk flag).

We estimated adjusted risk ratios (RRs) and the predicted probability (average marginal effects) of any video telehealth use in a month using generalized linear Poisson models with a log link function. Adjusted RRs can be interpreted as the relative likelihood of a video visit in a given month between months without a tablet and months with a tablet. We also estimated the adjusted incidence rate ratios (IRRs) for video telehealth and predicted the count of video visits per month (average marginal effects) using generalized linear negative binomial models with a log link function, which may be interpreted similarly. All models report 95% CIs and have standard errors clustered at the patient level.

#### Video-Based Services by Referral Reason in the Digital Divide Consult

To evaluate video-based services by referral reason in the Digital Divide Consult, we restricted the study population of 91,196 veterans who received a tablet after the Digital Divide Consult to 79,230 veterans who had a consult completed no later than 6 months before the tablet ship date and no more than 30 days after the tablet ship date (to account for delayed consult completion). We have reported the percentage of postconsult tablet recipients with any video visit. We have reported the unadjusted mean number of video visits during the first 6 months of tablet possession among the 79,230 veterans (including veterans with no or zero video visits) using *t*-tests. We estimated the adjusted mean number of video visits using a negative binomial model for each Digital Divide Consult reason, with adjustment only for age at tablet receipt, gender, race, ethnicity, and rurality of the home.

Finally, as a sensitivity analysis to identify if an increased number of consult reasons was associated with increased video use, we also evaluated the association between the number of consult reasons selected and the probability of a video visit and predicted number of video visits in a month using a Poisson model and a negative binomial model, respectively. This analysis was minimally adjusted for age at tablet receipt, race, ethnicity, gender, and rurality of the home.

All statistical analyses were conducted in Stata 18 (StataCorp, LLC).

### Ethical Considerations

This evaluation was conducted as part of the Virtual Access QUERI, which is designated as quality improvement by VA’s Office of Rural Health and VA Research Administration. The institutional review board at the Stanford Research Compliance Office determined this evaluation does not meet the requirement of research or clinical investigation per Federal Regulations 45CFR 46.104 [35] and VA 38CFR 16.104 [36].

## Results

### Nationwide Trends in Telehealth and Tablet Distribution in the Study Sample

**Figure 1:**
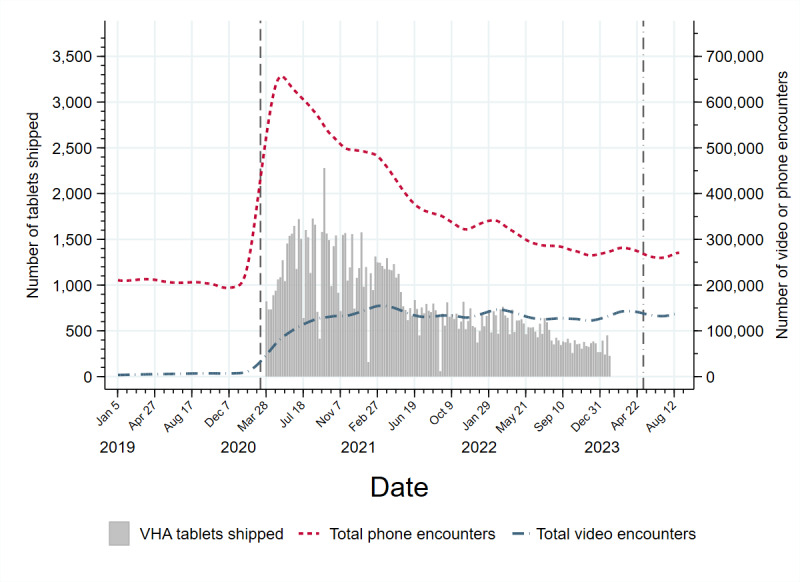
Smoothed weekly trends in video and phone visits and counts of weekly tablet distribution in the Veterans Health Administration (VHA) between January 5, 2019, and September 2, 2023. The vertical dashed line represents March 11, 2020 (WHO declares COVID-19 a pandemic). The vertical dash-dot line represents May 11, 2023 (end of federal COVID-19 public health emergency declaration).

A total of 119,926 veterans received a tablet between April 1, 2020, and February 28, 2023, representing just over 2% of veterans who are actively using VHA health care. VHA tablet distribution increased in line with the expansion of video and phone-based care in early 2020 following the start of the COVID-19 pandemic and decreased as the pandemic continued (Figure 1). The largest number of tablets was distributed in late summer and early fall of 2020, with a peak of 2280 tablets shipped the week of September 21, 2020 (Figure 1).

### Sociodemographic and Clinical Characteristics of Tablet Recipients and the General VHA Population

Similar to the general VHA population, veterans who received a tablet were predominately white, male, and living in urban areas (Table 2). Veterans who received a tablet were on average 2 years younger than the general population of veterans (mean age: 64 vs 66 years) and on average had 2 more chronic conditions (4 vs 2). The proportion of tablet recipients who were black was nearly twice the proportion among the general VHA population (34,901/119,926, 29.1% vs 121,666/683,219, 17.8%). Larger proportions of tablet recipients than the general VHA population were living in urban areas (81,985/119,926, 68.4% vs 447,235/683,219, 65.5%), were classified as low income (30,889/119,926, 25.8% vs 122,003/683,219, 17.9%) or high disability in VHA enrollment priority groups (59,744/119,926, 49.8% vs 279,530/683,219, 40.9%), or were single, divorced, or widowed (70,007/119,926, 58.4% vs 290,989/683,219, 42.6%). A smaller proportion of tablet recipients than the general VHA population were of Hispanic or Latino ethnicity (6139/119,926, 5.1% vs 47,468/683,219, 6.9%). Tablet recipients had twice the prevalence of a mental health condition (82,066/119,926, 68.4%), 3 times the prevalence of hospitalization (14,226/119,926, 11.9%), and over 4 times the prevalence of a recent history of housing instability (3361/119,926, 2.8%), homelessness (23,002/119,926, 19.2%), or a suicide flag in their health care records (3014/119,926, 2.5%) compared to the general VHA population (Table 2). Tablet recipients also had higher levels of prior engagement with the VHA health care system at baseline than the general VHA population (eg, higher proportion of any use of VHA outpatient visits across all modalities and care types) (Multimedia Appendix 2).

**Table 2:** Demographic and clinical characteristics of Veterans Health Administration (VHA) tablet recipients and a 5:1 probability sample of the general VHA population (N=803,145) active in VHA care.

Variable		General VHA^a^ population (N=683,219), n (%)	All tablet recipients (N=119,926), n (%)	Tablet recipients before Digital Divide Consult implementation (N=28,730), n (%)	Tablet recipients after Digital Divide Consult implementation (N=91,196), n (%)	
**Age (years)**						
	18-44	124,356 (18.2)	14,430 (12.0)	3189 (11.1)	11,241 (12.3)	
	45-64	200,718 (29.4)	48,199 (40.2)	10,556 (36.7)	37,643 (41.3)	
	≥65	358,145 (52.4)	57,297 (47.8)	14,985 (52.2)	42,312 (46.4)	
**Sex**						
	Male	619,267 (90.6)	104,890 (87.5)	25,492 (88.7)	79,398 (87.1)	
	Female	62,310 (9.1)	14,254 (11.9)	3067 (10.7)	11,187 (12.3)	
	Gender diverse	1642 (0.2)	782 (0.7)	171 (0.6)	611 (0.7)	
**Race**						
	Asian	6354 (0.9)	1212 (1.0)	289 (1.0)	923 (1.0)	
	American Indian or Alaska Native	8303 (1.2)	765 (0.6)	190 (0.7)	575 (0.6)	
	Black	121,666 (17.8)	34,901 (29.1)	7336 (25.5)	27,565 (30.2)	
	Native Hawaiian or Pacific Islander	6446 (0.9)	1069 (0.9)	281 (1.0)	788 (0.9)	
	Missing	46,719 (6.8)	6082 (5.1)	1434 (5.0)	4648 (5.1)	
	White	493,731 (72.3)	75,897 (63.3)	19,200 (66.8)	56,697 (62.2)	
**Ethnicity**						
	Not Hispanic or Latino	608,765 (89.1)	110,336 (92.0)	26,542 (92.4)	83,794 (91.9)	
	Hispanic or Latino	47,468 (6.9)	6139 (5.1)	1372 (4.8)	4767 (5.2)	
	Missing	26,986 (3.9)	3451 (2.9)	816 (2.8)	2635 (2.9)	
**Marital status**						
	Single, divorced, or widowed	290,989 (42.6)	70,007 (58.4)	16,580 (57.7)	53,427 (58.6)	
	Married	381,997 (55.9)	48,934 (40.8)	11,917 (41.5)	37,017 (40.6)	
	Unknown	10,233 (1.5)	985 (0.8)	233 (0.8)	752 (0.8)	
**Rural or urban status**						
	Urban	447,235 (65.5)	81,985 (68.4)	19,663 (68.4)	62,322 (68.3)	
	Rural	227,082 (33.2)	36,840 (30.7)	8825 (30.7)	28,015 (30.7)	
	Highly rural	8902 (1.3)	1101 (0.9)	242 (0.8)	859 (0.9)	
**Priority enrollment category**						
	No special enrollment	114,620 (16.8)	8589 (7.2)	2100 (7.3)	6489 (7.1)	
	Low income	122,003 (17.9)	30,889 (25.8)	7558 (26.3)	23,331 (25.6)	
	Low or moderate disability	167,066 (24.5)	20,704 (17.3)	4962 (17.3)	15,742 (17.3)	
	High disability	279,530 (40.9)	59,744 (49.8)	14,110 (49.1)	45,634 (50.0)	
**Drive time to the primary site of care (min)**						
	<30	533,772 (78.1)	96,786 (80.7)	23,586 (82.1)	73,200 (80.3)	
	≥30	141,575 (20.7)	22,332 (18.6)	4966 (17.3)	17,366 (19.0)	
	Unknown	7872 (1.2)	808 (0.7)	178 (0.6)	630 (0.7)	
**Number of chronic conditions**						
	0-3	470,628 (68.9)	48,221 (40.2)	10,355 (36.0)	37,866 (41.5)	
	4-6	175,048 (25.6)	49,865 (41.6)	12,299 (42.8)	37,566 (41.2)	
	7-9	32,350 (4.7)	17,660 (14.7)	4790 (16.7)	12,870 (14.1)	
	≥10	2544 (0.4)	2225 (1.9)	688 (2.4)	1537 (1.7)	
	Missing	2649 (0.4)	1955 (1.6)	598 (2.1)	1357 (1.5)	
Mental health condition		228,538 (33.5)	82,066 (68.4)	19,916 (69.3)	62,150 (68.1)	
History of hospitalization (in the 180 days before March 1, 2020)		25,692 (3.8)	14,226 (11.9)	3902 (13.6)	10,324 (11.3)	
Housing instability		4068 (0.6)	3361 (2.8)	860 (3.0)	2501 (2.7)	
Homelessness		25,809 (3.8)	23,002 (19.2)	6175 (21.5)	16,827 (18.5)	
High risk for suicide (any suicide electronic health care record flag in the 12 months before March 1, 2020)		3069 (0.4)	3014 (2.5)	725 (2.5)	2289 (2.5)	

^a^VHA: Veterans Health Administration.

### Evaluation of the Digital Divide Consult and Video Telehealth Use

The Digital Divide Consult was implemented in September 2020, but there was variation in the implementation by the VHA medical facility. By December 2020, 130 out of 139 (93.5%) VHA medical centers were using the Digital Divide Consult. Among all 139 VHA medical centers, 99 (71.2%) provided a Digital Divide Consult to ≥80% of tablet recipients during the last 3 months of 2020. A year after the Digital Divide Consult implementation, 131 (94%) VHA medical centers provided a Digital Divide Consult to ≥80% of tablet recipients. Among the 91,196 tablet recipients after consult implementation, 86.9% (n=79,230) had an identifiable Digital Divide Consult completed.

Among tablet recipients, those who received their tablets after consult implementation were largely similar to those who received their tablets before (Table 2). All veterans who received a tablet had nearly 3 times the likelihood of having a video visit in a month compared to the general VHA population, with an adjusted RR of 2.95 (95% CI 2.91-2.99) before consult implementation and 2.73 (95% CI 2.70-2.76) after consult implementation (Table 3). This corresponds to a predicted probability of having a video visit in a month of 0.21 (95% CI 0.20-0.21) and 0.19 (95% CI 0.19-0.19) among tablet recipients before and after consult implementation, respectively, and 0.07 (95% CI 0.069-0.090) among the general VHA population. Tablet recipients also had a higher predicted average count of video visits per month, at an IRR of 4.47 (95% CI 4.36-4.59) and 4.27 (95% CI 4.20-4.34) among tablet recipients before and after consult implementation, respectively, compared to the general VHA population. This corresponds to an average of 3 visits every 6 months among tablet recipients compared to less than 1 visit among the general VHA population.

**Table 3:** Monthly video visit comparing the general Veterans Health Administration population, tablet recipients before Digital Divide Consult implementation, and tablet recipients after Digital Divide Consult implementation.

Group^a^	Likelihood of any video visit per month^b^		Count of video visits per month^b^	
	Risk ratio (95% CI)	Predicted probability of a video visit (95% CI)	Incidence rate ratio (95% CI)	Predicted number of video visits per month (95% CI)
General VHA^c^ population	1.00 (reference)	0.07 (0.069-0.070)	1.00 (reference)	0.12 (0.12-0.12)
Tablet recipients before consult	2.95 (2.91-2.99)	0.21 (0.20-0.21)	4.47 (4.36-4.59)	0.55 (0.53-0.56)
Tablet recipients after consult	2.73 (2.70-2.76)	0.19 (0.19-0.19)	4.27 (4.20-4.34)	0.52 (0.52-0.53)

^a^For the tablet cohort, we report video visits in the first 7 months of tablet receipt but exclude the first month of tablet receipt from models as tablet assignment occurred in these months and we did not want to attribute tablet assignment-related visits to the tablet-associated outcome; therefore, we had a maximum of 6 months of follow-up after tablet receipt among tablet recipients.

^b^Models were adjusted for age at tablet receipt; gender; race; ethnicity; rurality of the home; VHA enrollment priority category; number of chronic conditions; presence of a mental health condition; current marital status; drive time to the closest primary VHA facility; any hospitalization in the 180 days before March 1, 2020; any suicide flag in the 12 months before March 1, 2020; history of housing instability; history of homelessness; history of video, phone, and remote patient monitoring in 2019; history of primary care, mental health, specialty care use, and other VHA care (diagnostic/ancillary care) in 2019; and VHA medical center. Each model also included fixed effects for the calendar month and standard errors accounted for clustering at the patient level.

^c^VHA: Veterans Health Administration.

In a secondary analysis assessing the use of phone care, veterans who received a tablet before the implementation of the Digital Divide Consult had 1.26 (95% CI 1.25-1.27) times the likelihood of having a phone-based visit, while veterans who received their tablet after consult implementation had a RR of 1.48 (95% CI 1.47-1.49) compared to the general VHA population (Multimedia Appendix 3). Veterans with tablets also had a higher predicted number of phone visits, with 0.73 (95% CI 0.72-0.74) phone visits per month among veterans who received their tablet before consult implementation and 0.83 (95% CI 0.83-0.84) phone visits per month among those who received their tablet after consult implementation in the first 6 months of tablet possession compared to 0.44 (95% CI 0.44-0.44) visits per month among the general VHA population.

### Video-Based Services by Referral Reason in the Digital Divide Consult

Among the 79,230 veterans who received a tablet with a Digital Divide Consult, the most common reason for receiving a tablet was having a mental health diagnosis (50,367/79,230, 63.6%), followed by living more than 30 miles away (17,228/79,230, 21.7%) and social isolation (16,161/79,230, 20.4%) (Table 4; Multimedia Appendix 4). The mean number of criteria checked was 1.97 (25th percentile-75th percentile: 1-3). Approximately 5407 veterans received a tablet with a consult stating they met no specific criteria or with no consult reason selected. A manual review of these consults highlighted additional reasons that veterans were referred for tablets, including avoiding health care facilities due to COVID-19, perceptions that a person might benefit from a device to increase patient-provider communication, and enrollment in a VHA health care program that relies on virtual appointments such as the VHA MOVE weight loss program.

On average, 63.0% (49,925/79,230) of individuals who received a tablet with a Digital Divide Consult had a video visit in the first 6 months of tablet receipt. Some Digital Divide Consult reasons were associated with a higher proportion of tablet recipients using video telehealth (Table 4). These reasons included being enrolled in evidence-based mental health programs (74.8% [830/1100] with any video use), living more than 30 miles away from a VHA center (68.3% [10,557/17,228] with any video use), having a mental health diagnosis (68.1% [34,301/50,367] with any video use), and finding in-person visits challenging (65.1% [4215/6472] with any video use). Other reasons were associated with a lower proportion of tablet recipients using video telehealth, such as disruptive behavior (56.9% [230/404] with any video use), being in hospice (55.2% [219/397] with any video use), and not meeting any of the specific criteria at the consult (58.4% [3159/5407] with any video use).

The mean number of video visits in the first 6 months of tablet receipt also varied by consult reason selected (Table 4). Consult reasons associated with a higher adjusted mean number of video visits per month included evidence-based mental health (7.58), social isolation (5.44), and any mental health condition (5.33). The mean number of visits in the first 6 months of tablet receipt among tablet recipients with no criteria selected was 4.23 (95% CI 3.98-4.48), and the mean number of visits with any of the criteria selected was 4.77 (95% CI 4.69-4.84) (*t*-test for difference *P*<.01). As the mean numbers can be influenced by outliers of video telehealth use, we also describe the proportion of tablet recipients having no visits, 1-3 visits, and 4 or more visits in a 6-month period in Multimedia Appendix 5. There was no relationship between the probability of having a video visit in the first 6 months and the number of consult reasons selected; however, there was a slightly increasing linear relationship between the number of consult reasons selected and the predicted number of video visits in a month (Multimedia Appendix 6).

**Table 4:** Percentage of Veterans Health Administration tablet recipients with any video use and mean number of video visits by consult reason (Digital Divide Consult) (N=79,230).

Digital Divide Consult reason^a^	Tablet recipients reporting a given Digital Divide Consult reason, n (%)	Percentage of tablet recipients with any video use^b^, n (%)	Unadjusted mean number of video visits^c^ in 6 months by reason reported, value (95% CI)	Adjusted mean number of video visits^d^ in 6 months by reason reported, value (95% CI)
Enrolled in evidence-based mental health^e^	1100 (1.4)	830 (74.8)	8.60 (7.82-9.39)	7.58 (7.09-8.09)
Lives more than 30 miles from a VHA facility^f^	17,228 (21.7)	10,557 (68.3)	4.01 (3.89-4.14)	4.53 (4.45-4.62)
Any mental health diagnosis	50,367 (63.6)	34,301 (68.1)	5.70 (5.61-5.80)	5.33 (5.28-5.39)
Work, school, or caregiver commitments make in-person visits challenging	6472 (8.2)	4215 (65.1)	4.62 (4.40-4.84)	4.33 (4.21-4.45)
Homeless veteran or enrolled in the HUD-VASH^g^ Program	6434 (8.1)	3988 (62.0)	4.97 (4.72-5.21)	4.21 (4.10-4.33)
Social isolation	16,161 (20.4)	10,016 (62.0)	5.24 (5.07-5.41)	5.44 (5.34-5.53)
Difficulty attending a VHA facility (ie, immunocompromised state or psychological distress)	13,324 (16.8)	8165 (61.3)	4.39 (4.23-4.55)	4.66 (4.57-4.75)
Cost of attending in-person is prohibitive	4608 (5.8)	2809 (61.0)	4.68 (4.40-4.96)	4.74 (4.68-4.89)
Hospitalized in the last 90 days	6401 (8.1)	3823 (59.7)	5.01 (4.74-5.23)	4.87 (4.74-5.01)
No specific criteria or no criteria selected	5407 (6.8)	3159 (58.4)	4.23 (3.98-4.48)	4.40 (4.27-4.54)
Health issues make the veteran homebound	13,280 (16.8)	7739 (58.3)	3.21 (3.09-3.33)	4.13 (4.04-4.22)
Difficulty with public transportation	9354 (11.8)	5383 (57.6)	3.79 (3.61-3.98)	4.28 (4.18-4.38)
No car or access to a ride	7983 (10.1)	4575 (57.3)	4.36 (4.13-4.59)	4.30 (4.19-4.41)
No veteran transport or DAV^h^	2743 (3.5)	1563 (57.0)	3.88 (3.56-4.21)	4.23 (4.05-4.42)
Documented disruptive behavior at VHA	404 (0.5)	230 (56.9)	3.99 (3.15-4.82)	3.56 (3.17-3.96)
Veteran has a hospice diagnosis^i^	397 (0.5)	219 (55.2)	3.52 (2.76-4.28)	3.87 (3.43-4.31)

^a^More than one criterion could be selected (mean number of criteria checked was 1.97 [25th percentile-75th percentile: 1-3]).

^b^Results are sorted by ranked percentage of tablet recipients with any video use.

^c^Unadjusted mean number of video visits was calculated using the *t*-test.

^d^The adjusted mean number of video visits is the average marginal effect where the independent variable was the selection of one of the Digital Divide Consult reasons (ie, reasons were not mutually adjusted for) and the dependent variable was the count of video visits (including veterans with no or zero video visits). Models were adjusted for age at tablet receipt, gender, race, ethnicity, and rurality of the home.

^e^Analytic sample for the “evidence-based mental health” consult reason has been restricted to veterans who received their tablets after March 1, 2020, when this reason was added (N=17,746).

^f^VHA: Veterans Health Administration.

^g^HUD-VASH: US Department of Housing and Urban Development-VA Supportive Housing.

^h^DAV: Disabled American Veterans.

^i^Analytic sample for the “hospice” consult reason has been restricted to veterans who received their tablets after May 1, 2021, when this reason was added (N=43,933).

## Discussion

Since 2016, VHA’s Connected Device Program has provided tablets to over 180,000 veterans. To our knowledge, this unique program was the first of its size in the United States to bridge the digital divide by offering patients a device to engage in video telehealth use. Findings from this evaluation suggest that the program has reached many veterans from groups that have experienced health care access disparities, including women veterans, gender-diverse veterans, veterans who are black, veterans who have a history of homelessness, and those with low-income priority status in VHA. While these patient characteristics are not explicitly targeted as a criterion in the Digital Divide Consult, supporting veterans who have historically faced health care access barriers and disparities aligns with VHA’s Office of Health Equity. However, the underuse of tablets (only 63% of recipients after the Digital Divide Consult had video use within 6 months) indicates an opportunity for additional implementation strategies to ensure that those who receive this resource have the support they need to engage in video telehealth use.

In this evaluation, 68.4% (81,985/119,926) of tablet recipients lived in urban areas compared to a previous evaluation, where 47% of tablet recipients between May 1, 2016, and September 30, 2017, lived in urban areas [13]. This shift is likely a result of the COVID-19 pandemic’s restriction on in-person care and the expansion of video-based care availability in multiple care services [9,37,38]. An evaluation of the characteristics of tablet recipients before and during the COVID-19 pandemic found that 55% of prepandemic tablet recipients were urban-dwelling compared with 68% during the pandemic but before the implementation of the Digital Divide Consult, which closely matches our result of 68.4% [15].

This evaluation also found that the tablet population has become more racially diverse, older, and more likely to be single compared with earlier evaluations [13,15]. This may be a reflection of the broadening of eligibility reasons for a tablet receipt in the Digital Divide Consult. Between 2016 and 2017, the eligibility criteria for access barriers included only distance or geography, transportation issues, difficulty leaving home, and other reasons described by the provider [13]. The increased tablet allotment to veterans with a history of hospitalization, a suicide risk flag, a history of mental health conditions, and a disability-related priority status suggests that tablets are directed to veterans with complex medical needs as intended and may be a direct result of the inclusion of these complex medical needs as a criterion for tablet receipt. Interestingly, the most common reason for tablet recipients was the presence of a mental health condition. This increased distribution of tablets among this population may be a consequence of a higher uptake of video-based care among mental health service providers [38].

The Digital Divide Consult was designed by OCC as a mechanism to standardize screening and referrals for VHA-issued tablets and internet service [39]. This consult improved the device-lending process by introducing a social worker assessment to identify and resolve barriers to care such as internet connectivity needs. We found that tablet receipt was associated with elevated likelihoods and rates of using video telehealth. Yet, there was a small but statistically significant decrease in the likelihood and rate of video telehealth use before versus after implementation of the Digital Divide Consult. Tablet recipients before the consult had 2.95 times the likelihood of a video visit and 4.47 times the incidence rate compared with the general VHA population in the same month. The RR of 2.73 and IRR of 4.27 were slightly smaller among tablet recipients after the consult compared with the general VHA population in the same month. While we adjusted for the calendar month in our analysis and made comparisons within a given month, we may not have fully accounted for temporal differences such as video care availability in VHA across preconsult and postconsult months. As seen in Figure 1, the preconsult period of April 1, 2020, to October 1, 2020, was marked by abnormally high availability of video care. In comparison, the postconsult period of October 1, 2020, through the end of follow-up, August 31, 2023, was marked by a reduction in video care and an increase in in-person care availability, establishing the new equilibrium of telemedicine following the COVID-19 pandemic [38]. Even so, the difference between pre- and postconsult tablet recipients is likely clinically nonsignificant as it translates to a difference of an additional visit every 3 years, assuming the rate of visits remains the same over time.

Veterans who received a tablet also had increased engagement in phone visits compared to the general VHA population, suggesting that tablets may increase veteran engagement with VHA beyond video visits through increased patient-provider communication as tablets are not set up to complete phone calls. This is consistent with prior work that demonstrated providing tablets to veterans also increased engagement in health information and management tasks in the veterans’ My Health*e*Vet patient portal [40].

Despite the successes of VHA’s Connected Device Program, challenges remain. The rate of video visit use among tablet recipients is lower than initially expected, and this pattern has been observed in tablet distribution programs outside VHA [41], suggesting a need for interventions to ensure that all veterans who receive VHA-issued tablets are able to use them [17]. In 2021, OCC launched the Connected Devices Support Program to build digital literacy among recipients of VHA-issued tablets. As a part of this program, a technician offers device education to veteran recipients of loaned tablets and a video test call ahead of the first video visit with their VHA care team. Other findings, such as the observed variation in video visit use among veterans who met specific criteria for a tablet, can inform implementation strategies within clinical services. For example, veterans who were referred for tablets to participate in a specific treatment protocol (ie, evidence-based mental health) were much more likely to have a subsequent video visit than those who were referred for more general reasons such as transportation barriers. This suggests that coupling tablet distribution with a specific treatment program or service might increase the likelihood of tablet use. The increased predicted number of video visits among evidence-based mental health and mental health–related consult reasons may reflect the underlying treatment protocols. For example, mental health visits in programs like the Intensive Community Mental Health Recovery (ICMHR) or Substance Use Disorder Intensive Outpatient Programs occur at a high intensity, corresponding to an average frequency of 7 to 15 visits per month [42], while primary care visits occur much less frequently, leading to a differential predicted number of visits by consult reasons.

Several limitations should be considered when interpreting these findings. First, the analysis does not provide information on whether video visits occurred specifically through the tablets. As veterans were screened for the possession of a video-enabled device, we assume that a subsequent video visit occurred using the VHA-provided tablet. Moreover, the study’s focus on video may have overlooked other activities that could potentially contribute to veterans’ health care and health navigation, such as their interaction with the VHA’s patient portal and My HealtheVet, or the use of various health-related mobile apps. Additional research from the Virtual Access QUERI indicates that tablets increase engagement in patient portals, which may reduce the need for a subsequent visit (eg, medication management requests through messaging rather than an appointment).

Second, our results may be limited as the general veteran population may be different from veterans who received a tablet and, more specifically, those who received a tablet through the Digital Divide Consult (ie, exchangeability assumption). In our analysis, we accounted for differences between the 2 populations by identifying and adjusting for key patient demographics associated with the differential use of video telehealth (eg, age, history of VHA use, and rurality of the home) [10] and characteristics similar to those captured in the Digital Divide Consult. We identified electronic health record equivalents for Digital Divide Consult reasons such as drive time to a VHA facility, mental health conditions, history of hospitalization, housing instability, history of homelessness, and suicide risk. We were unable to identify some Digital Divide Consult reasons that referred to social needs as these are not captured in the current electronic health record (eg, social isolation, difficulty attending a VHA facility, cost of attending is prohibitive, and no car or access to VHA). Thus, our results may be biased by nonexchangeability between the study groups. An alternative to our statistical adjustment would be emulating multiple nested trials, with each having a different start of follow-up (n=40 for each calendar month for tablet receipt), which is complicated to implement [43].

Additionally, our choice of the baseline period for veteran demographics and characteristics may influence our results. We chose a baseline period prior to the time of the first tablet receipt for the entire population, which may be less accurate for tablet recipients in the later years of our evaluation. However, most of these variables, such as race, rurality, and gender, are unlikely to change over the course of the study. However, 4 variables in our analysis represent more acute and temporally relevant windows. Changes in these variables, such as a new occurrence of suicide risk or homelessness, which may have increased in the acute phase of the pandemic, may bias our adjustment of baseline differences in use between tablet recipients and the general VHA population. Despite this, our approach is relatively robust as we compared general trends in patient demographics and video care use and as the sample size of the comparator population was sufficiently large (temporal variations in these 4 confounding variables will not unduly influence our analysis). This is a limitation of our approach and of observational data in general when eligibility criteria for the population of interest are met at multiple times [29].

In prior evaluations of the Connected Devices Program, statistical matching on patient characteristics has been used to compare the use of VHA health care among tablet recipients with more similar veterans from the general VHA population [18,19]. In this evaluation, matching was not used, as one of our primary questions was to compare the distribution of population characteristics among tablet recipients with the general VHA population. Matching these patient characteristics would prevent us from comparing these distributions between the 2 groups in our first objective.

Finally, we considered veterans who received tablets after the implementation of the Digital Divide Consult as part of the “postdigital divide” group regardless of whether there was an associated Digital Divide Consult for each patient. Among the postconsult group, 90% of the consults were identified, leading to an assumption of an intent-to-treat framework, which may result in underestimating the actual relationship between Digital Divide Consult implementation and the use of video or phone care. The frequency of tablet recipients after consult with an identifiable consult increased with time, indicating a growing use of the Digital Divide Consult. These limitations should be considered when interpreting the study’s findings and their implications for health care policy and practice.

In summary, the VHA’s Digital Divide Consult offers information about why veterans receive tablets and the degree to which different subgroups, who are often left out of technology initiatives, may use tablets. Findings from this evaluation suggest that VHA’s tablet distribution effort is reaching veterans from a number of high-need groups and has likely facilitated the use of video telehealth. Ongoing evaluation activities are examining the cost implications of VHA’s Connected Device Program, the experiences of tablet recipients who do not use or who underuse their tablets, and the use of tablets for engagement in health care outside of video visits. With the expansion of video telehealth as a modality for health care delivery, VHA’s Connected Device Program can serve as a model for other health care systems that aim to expand access for individuals who lack their own devices.

## Appendix

Multimedia Appendix 1 Detailed sampling methodology and description of variable composition and definition.

Multimedia Appendix 2 History of Veterans Health Administration (VHA) outpatient health care use (March 11, 2019, to March 10, 2020) among tablet recipients and the general VHA population.

Multimedia Appendix 3 Monthly phone visit use comparing the general Veterans Health Administration population, tablet recipients before Digital Divide Consult, and tablet recipients after Digital Divide Consult.

Multimedia Appendix 4 Prevalence of the reasons for the Digital Divide Consult, ordered by frequency selected, among 79,230 tablet recipients. Multiple criteria can be selected for one consult. Commitments that make in-person visits challenging include school, work, or caregiver commitments. Difficulty attending VHA included reasons like immunocompromised state or psychological distress. Hospice consult and evidence-based consult reasons were added in May 1, 2021, and March 1, 2020, respectively. DAV: Disabled American Veterans; HUD-VASH, US Department of Housing and Urban Development-Veterans Affairs Supportive Housing; VHA: Veterans Health Administration.

Multimedia Appendix 5 Mean count of video visits among veterans with a Digital Divide Consult (N=79,230) by consult reason.

Multimedia Appendix 6 Adjusted association between the number of consult reasons selected and the probability of any video use and change in the predicted number of video visits in a month among veterans with a Digital Divide Consult (N=79,230).

## Abbreviations

CDW: Corporate Data Warehouse
IRR: incidence rate ratio
OCC: Office of Connected Care
ORH: Office of Rural Health
QUERI: Quality Enhancement Research Initiative
RR: risk ratio
VHA: Veterans Health Administration

## Data Availability

Owing to US Department of Veterans Affairs (VA) regulations and our ethics agreements, the analytic data sets used for this study are not permitted to leave the VA security system without a data use agreement. This limitation is consistent with other studies based on VA data. However, VA data are made freely available to researchers with an approved VA study protocol. For more information, please visit [44] or contact the VA Information Resource Center at VIReC@va.gov.
