# Supplementary material for: Evaluation of the Veterans Health Administration’s Digital Divide Consult for Tablet Distribution and Telehealth Adoption: Cohort Study
Source: J Med Internet Res. 2024 Sep 9;26:e59089. doi: 10.2196/59089 (PMC11420580; doi:10.2196/59089)
Supplement: Multimedia Appendix 1 [file jmir_v26i1e59089_app1.docx]

**Multimedia Appendix 1.** Detailed sampling methodology and description of variable composition and definition.

**Detailed sampling methodology for analytic cohort**We selected our study sample from an existing cohort of over 5.3 million Veterans who were actively engaged in care in 2019. (Ferguson JM, Wray CM, Greene L, et al. Variation in initial and continued use of primary, mental health, and specialty video care among Veterans. *Health Serv Res*. 2022;58(2):402-414. doi:10.1111/1475-6773.14098). Active engagement in VHA care was defined as a Veteran assigned to a primary care management team and who had at least one VHA outpatient visit (excluding compensation or pension examinations) between March 11, 2019 and March 10, 2020: the day prior to COVID-19 being declared a pandemic by the World Health Organization.

From within this parent cohort, we identified 125,784 Veterans who received a tablet between April 2020 and February 2023, using the most recent tablet shipment date. To balance computational load and statistical efficiency, we selected 700,000 Veterans who had not received a tablet using a random sample where all non-tablet recipients had an equal chance of inclusion. A 5:1 sampling framework was chosen as statistical power sharply increases with an increasing sampling ratio until 1:4 or 1:5 and then slowly increases thereafter (Hennessy S, Bilker WB, Berlin JA, Strom BL. Factors influencing the optimal control-to-case ratio in matched case-control studies. Am J Epidemiol 1999; 149: 195–197.).

To ensure we selected a representative sample, we repeated this sampling procedure five times and chose the subsample whose demographic and clinical characteristics best approximated the 5.2 million parent sample. From this cohort of 825,784 tablet and non-tablet recipients Veterans, we excluded Veterans whose parent facility had recently adopted a new electronic healthcare record system CERNER (limited data availability) and those had less than 1 month of follow-up (non-tablet recipients those who died in April 2020 and tablet recipients those who died within 1 month of tablet receipt). Veterans who were missing data on their home zip code (required for rural/urban classification) and priority enrollment group were excluded from analysis (<0.5% combined). This left a final sample of 119,926 tablet recipients and 683,219 randomly selected Veterans from the general population.

**History of housing instability, history of homelessness**
History of housing instability and history of homelessness were defined using ICD10 diagnostic codes, VHA outpatient stop codes, VHA inpatient specialty codes, and health factors from the Homeless Screening Clinical Reminder (HSCR) following the methodology from Tsai et al. 2022. Participation in the Homeless Management Information System (HMIS) and the Homeless Operations, Management, and Evaluation System (HOMES) were not available for the creation these indicators. Rural and urban designations among these Veterans may reflect VHA domiciliary addresses, VA and U.S Department of Housing and Urban Development supportive housing, or their last known physical address.

Reference: Tsai J, Szymkowiak D, Jutkowitz E. Developing an operational definition of housing instability and homelessness in Veterans Health Administration’s medical records. *PLoS One*. 2022;17(12). doi:10.1371/journal.pone.0279973

**Drive time**
Drive time distances to patients’ closest facilities were obtained from the VA’s Planning Systems Support Group.

**List of chronic conditions**
Twenty-eight chronic conditions and diagnoses were defined using International Statistical Classification of Disease (ICD) 10 codes, selected based on prior VA research. Chronic conditions include: Acid Related Diseases, Cancers (all types), Alzheimer’s Disease, Arthritis, Asthma, Chronic Obstructive Pulmonary Disease, Heart Failure, Diabetes, HIV/AIDS, Headache, Hepatitis C, Hypertension, Ischemic Heart Diseases, Lower Back Pain, Multiple Sclerosis, Parkinson’s Disease, Peripheral Vascular Disease, Pneumonia, Prostatic Hyperplasia, Renal Failure, Spinal Cord Injury, Stroke, Dementia, Traumatic Brain Injury.

References:
Sachs JW, Graven P, Gold JA, Kassakian SZ. Disparities in telephone and video telehealth engagement during the COVID-19 pandemic. JAMIA Open. 2021;4(3):1-5. doi:10.1093/jamiaopen/ooab056

Chunara R, Zhao Y, Chen J, et al. Telemedicine and healthcare disparities: a cohort study in a large healthcare system in New York City during COVID-19. J Am Med Inform Assoc. 2021;28(1):33-41. doi:10.1093/jamia/ocaa217

Eberly LA, Kallan MJ, Julien HM, et al. Patient Characteristics Associated With Telemedicine Access for Primary and Specialty Ambulatory Care During the COVID-19 Pandemic. JAMA Netw open. 2020;3(12):e2031640. doi:10.1001/jamanetworkopen.2020.31640

**List of mental health conditions**

Mental Health conditions were defined using VA Program Evaluation Resource Center

definitions and include substance use disorders, severe mental illness (bipolar disorder, severe

depression, and additional psychotic disorders), depression, and post-traumatic stress disorder

(PTSD).

Reference:
Fishman P, Von Korff M, Lozano P, Hecht J. Chronic Care Costs in Managed Care. *Health Aff*. 1997;16(3):239-247. doi:10.1377/hlthaff.16.3.239

**Description of VHA priority enrollment group**In our evaluation, we use a variable from VHA enrollment records called the VHA priority group. We defined Veterans with high disability as those in groups 1 (>50% service-connected disability) and 4 (VHA catastrophically disabled). Veterans with low/moderate disability include groups 2 (30-40% service-connected disability), 3 (10-20% service-connected disability, discharged due to disability), and 6 (military exposures and recent combat Veteran). Veterans with low-income include those in group 5 (annual income below area-adjusted income threshold). Finally, Veterans with no special enrollment considerations included Veterans from group 7 and group 8 (0% service-connected disability; agree to pay co-pays).

This variable may be introducing some misclassification bias to the analysis as Veterans are enrolled into the highest group (1 is highest) they qualify for resulting in non-mutually exclusive categorization. For example, because the low-income group is group 5 and disability rating place a Veteran in groups 4 or higher, Veterans who have a larger than a 10% service rated disability will be classified in a higher priority group regardless of their income status. Due to this ranking, the low-income category will not contain all Veterans who are low-income, but will only contain Veterans with low-income. Additionally, Veterans with military honors (e.g., Purple Heart or Medal of Honor) are placed in the highest two priority groups regardless of their disability or income rating which may introduce measurement error and bias when examining disability. The reference group for priority group are Veterans who do not meet any of the criteria for inclusion in the higher ranked groups. Therefore, the rates of the low-income group and disability categories will be attenuated towards the null and may underestimate the true association.

Reference for Area-adjusted income threshold: Wang JZ, Dhanireddy P, Prince C, Larsen M, Schimpf M, Pearman G. *2019 Survey of Veteran Enrollees’ Health and Use of Health Care- Data Findings Report*.; 2019. https://www.va.gov/HEALTHPOLICYPLANNING/SOE2019/2019_Enrollee_Data_Findings_Report-March_2020_508_Compliant.pdf

**Suicide flag description**

Patient record flags are used to alert VHA medical staff and employees of patients whose behavior and characteristics may pose a threat either to their safety, the safety of other patients, or compromise the delivery of quality health care. The high risk of suicide patient flag is a marker on the electronic health record designed to draw clinicians and providers to a patient whose behavior, medical status, or characteristics may pose an immediate threat to either that patient’s safety or others. The high risk of suicide flag is used when a patient has been identified as a high acute risk on a Comprehensive Suicide Risk Evaluation (CSRE), has had a recent suicide attempt or suicidal preparatory behavior (where recent is determined at a facility level), has been admitted for hospitalization due to suicidal ideation with intent, or is not able to maintain safety without external support.
