# Supplementary material for: Evaluation of the Veterans Health Administration’s Digital Divide Consult for Tablet Distribution and Telehealth Adoption: Cohort Study
Source: J Med Internet Res. 2024 Sep 9;26:e59089. doi: 10.2196/59089 (PMC11420580; doi:10.2196/59089)
Supplement: Multimedia Appendix 2 [file jmir_v26i1e59089_app2.docx]

**Multimedia Appendix 2.** History of Veterans Health Administration (VHA) outpatient health care use (March 11, 2019, to March 10, 2020) among tablet recipients and the general VHA population.

|  | Total | | General Population | | Tablet Recipients | |
| --- | --- | --- | --- | --- | --- | --- |
| **Baseline Use** | N=803,145 |  | N=683,219 |  | N=119,926 |  |
| **Video Visits Use** |  |  |  |  |  |  |
| None | 782,977 | 97.5% | 667,378 | 97.7% | 115,599 | 96.4% |
| Any | 20,168 | 2.51% | 15,841 | 2.3% | 4,327 | 3.6% |
| **Phone Use** |  |  |  |  |  |  |
| None | 281,759 | 35.1% | 262,766 | 38.5% | 18,993 | 15.8% |
| Any | 521,386 | 64.9% | 420,453 | 61.5% | 100,933 | 84.2% |
| **Remote Patient Monitoring** |  |  |  |  |  |  |
| None | 671,261 | 83.6% | 580,886 | 85.0% | 90,375 | 75.4% |
| Any | 131,884 | 16.4% | 102,333 | 15.0% | 29,551 | 24.6% |
| **Primary Care Use** |  |  |  |  |  |  |
| Low (0-1) | 161,183 | 20.1% | 149,609 | 21.9% | 11,574 | 9.7% |
| Middle (2-4) | 388,096 | 48.3% | 341,278 | 50.0% | 46,818 | 39.0% |
| High (5+) | 253,866 | 31.6% | 192,332 | 28.2% | 61,534 | 51.3% |
| **Mental Health Care Use** |  |  |  |  |  |  |
| Low/ Middle (0-1) | 551,409 | 68.7% | 507,979 | 74.4% | 43,430 | 36.2% |
| High (2+) | 251,736 | 31.3% | 175,240 | 25.6% | 76,496 | 63.8% |
| **Specialty Care Use** |  |  |  |  |  |  |
| Low (0) | 216,971 | 27.0% | 198,722 | 29.1% | 18,249 | 15.2% |
| Middle (1-6) | 348,931 | 43.4% | 306,363 | 44.8% | 42,568 | 35.5% |
| High (7+) | 237,243 | 29.5% | 178,134 | 26.1% | 59,109 | 49.3% |
| **Diagnostic/Ancillary Care** |  |  |  |  |  |  |
| Low (1-2) | 186,999 | 23.3% | 173,869 | 25.4% | 13,130 | 10.9% |
| Middle (3-9) | 386,508 | 48.1% | 340,342 | 49.8% | 46,166 | 38.5% |
| High (10+) | 229,638 | 28.6% | 169,008 | 24.7% | 60,630 | 50.6% |

For definitions on how care modality and care types were defined please reference prior work:

Ferguson JM, Jacobs J, Yefimova M, Greene L, Heyworth L, Zulman DM. Virtual care expansion in the Veterans Health Administration during the COVID-19 pandemic: clinical services and patient characteristics associated with utilization. J Am Med Informatics Assoc 2021;28(1):453–462. PMID:33125032

Ferguson JM, Wray CM, Greene L, Wagner TH, Odden MC, Freese J, Van Campen J, Asch SM, Heyworth L, Zulman DM. Variation in initial and continued use of primary, mental health, and specialty video care among Veterans. Health Serv Res 2022;58(2):402–414. PMID:36345235
