# Supplementary material for: Evaluation of the Veterans Health Administration’s Digital Divide Consult for Tablet Distribution and Telehealth Adoption: Cohort Study
Source: J Med Internet Res. 2024 Sep 9;26:e59089. doi: 10.2196/59089 (PMC11420580; doi:10.2196/59089)
Supplement: Multimedia Appendix 3 [file jmir_v26i1e59089_app3.docx]

**Multimedia Appendix 3.** Monthly phone visit use comparing the general Veterans Health Administration population, tablet recipients before Digital Divide Consult, and tablet recipients after Digital Divide Consult.

|  | **Likelihood of any  Phone Visit per month** | | **Count of Phone Visits  per month** | |
| --- | --- | --- | --- | --- |
|  | Risk Ratio  (95% CI) | Predicted probability of  phone use (95% CI) | Incidence Rate Ratio (95% CI) | Predicted number of phone visits per month (95% CI) |
| General VHA population | 1.00 (ref) | 0.24  (0.24, 0.24) | 1.00 (ref) | 0.44  (0.44, 0.44) |
| Tablet recipients before Digital Divide Consult  Implementation | 1.26  (1.25, 1.27) | 0.30  (0.30, 0.31) | 1.66  (1.64, 1.68) | 0.73 (0.72, 0.74) |
| Tablet recipients after Digital Divide Consult Implementation | 1.48  (1.47, 1.49) | 0.36  (0.36, 0.36) | 1.89  (1.87, 1.91) | 0.83 (0.83, 0.84) |

Models were adjusted for age at tablet receipt, gender, race, ethnicity, rurality of home, VHA enrollment priority category, Number of Chronic conditions, presence of a mental health condition, current marital status, drive time to closest primary VHA facility, any hospitalization in the 180 days before March 1, 2020, any suicide flag in the 12 months before March 1, 2020, history of housing instability, history of homelessness, history of video, phone, and remote patient monitoring in 2019, history of primary care, mental health, and specialty care use in 2019, and VHA medical center. Each model also included fixed effects for calendar month, and standard errors accounted for clustering at the patient-level. We assessed tablet use in the first 7 months of tablet receipt, but excluded the first month of tablet receipt from models as tablet assignment occurred in these months and we did not want to attribute tablet assignment-related visits to the tablet associated outcome, therefore we had a maximum of 6 months of follow-up after tablet receipt among Tablet Recipients.
