# Supplementary figures and images for: Evaluation of the Veterans Health Administration’s Digital Divide Consult for Tablet Distribution and Telehealth Adoption: Cohort Study
Source: J Med Internet Res. 2024 Sep 9;26:e59089. doi: 10.2196/59089 (PMC11420580; doi:10.2196/59089)

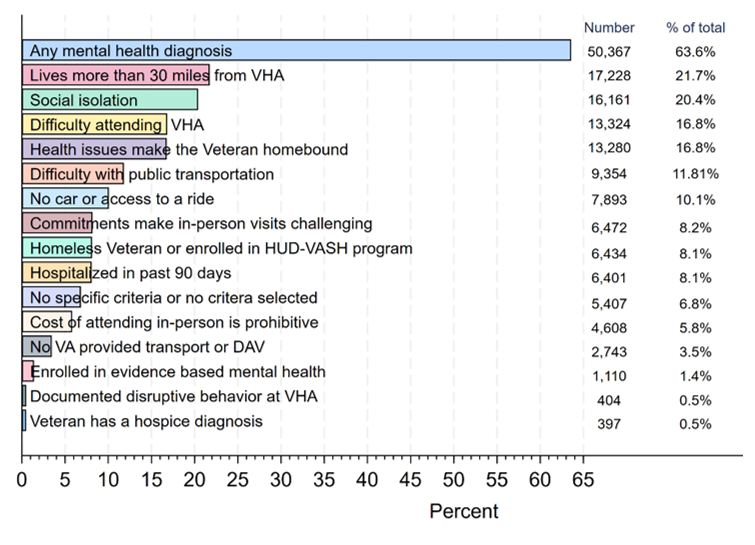

Supplement: Multimedia Appendix 4 [file jmir_v26i1e59089_app4.png]
