# Supplementary material for: Evaluation of the Veterans Health Administration’s Digital Divide Consult for Tablet Distribution and Telehealth Adoption: Cohort Study
Source: J Med Internet Res. 2024 Sep 9;26:e59089. doi: 10.2196/59089 (PMC11420580; doi:10.2196/59089)
Supplement: Multimedia Appendix 5 [file jmir_v26i1e59089_app5.docx]

**Multimedia Appendix 5.** Mean count of video visits among veterans with a Digital Divide Consult (N=79,230) by consult reason.

|  |  | **Video Visits in 6 months by reason reported** | | |
| --- | --- | --- | --- | --- |
| **Digital Divide Consult Reasons**† | **Total  N Veterans** | **No Video Visits** | **1-3 Video Visits** | **4 or more Video Visits** |
|  |  | Row % | Row % | Row % |
| Evidence based mental health* | 1,053 | 25.5 | 24.0 | 50.5 |
| Any Mental Health Diagnosis | 50,367 | 31.9 | 32.1 | 36.0 |
| In person visits are challenging | 6,472 | 34.9 | 32.8 | 32.3 |
| Homeless | 6,434 | 38.0 | 29.8 | 32.2 |
| Social isolation | 16,161 | 38.0 | 29.9 | 32.1 |
| Cost of attending is prohibitive | 4,608 | 39.0 | 30.1 | 30.8 |
| Difficulty attending VHA facility | 13,324 | 38.7 | 32.0 | 29.9 |
| Hospitalized in last 90 days | 6,407 | 40.3 | 30.3 | 29.5 |
| Lives more than 30 miles | 17,228 | 38.7 | 33.4 | 27.9 |
| No specific criteria/no criteria | 5,407 | 41.6 | 30.9 | 27.5 |
| No car or other ride | 7,983 | 42.7 | 30.7 | 26.6 |
| Disruptive Behavior | 404 | 43.1 | 31.0 | 26.0 |
| Hospice± | 391 | 45.3 | 29.2 | 25.6 |
| No VA transit (DAV) | 2,743 | 43.0 | 31.9 | 25.1 |
| Difficulty with public transportation | 9,354 | 42.5 | 32.6 | 24.9 |
| Homebound | 13,280 | 41.7 | 34.4 | 23.8 |
|  |  |  |  |  |
| Any Criteria (excludes Veterans with the no specific criteria/no criteria selected) | 73,823 | 36.7 | 32.5 | 30.9 |

Multiple criteria could be selected on one consult. Criteria are ordered according to largest to smallest percentage in the 4 or more-video visit column.

±Analytic sample for the ‘Hospice’ consult reason is restricted to Veterans who received their tablet after May 01, 2021, when this reason was added. N=43,933.

*Analytic sample for ‘evidence-based mental health’ consult reason is restricted to Veterans who received their tablet after March 01 2020, when this reason was added. N=17,746

† On a Veteran’s Digital Divide consult, multiple reasons can be selected. More than one criterion could be selected (mean number of criteria checked was 1.97 (P25- P75: 1-3). The median number of video visits was 1 for all consult reasons except for no specific criteria/no criteria (p50=0), Evidence based mental health (p50=4), and mental health diagnosis (p50=2); reflecting the highly skewed distribution of these data.
