# Supplementary material for: Evaluation of the Veterans Health Administration’s Digital Divide Consult for Tablet Distribution and Telehealth Adoption: Cohort Study
Source: J Med Internet Res. 2024 Sep 9;26:e59089. doi: 10.2196/59089 (PMC11420580; doi:10.2196/59089)
Supplement: Multimedia Appendix 6 [file jmir_v26i1e59089_app6.docx]

**Multimedia Appendix 6.** Adjusted association between the number of consult reasons selected and the probability of any video use and change in the predicted number of video visits in a month among veterans with a Digital Divide Consult (N=79,230).

| **Number of Consult reasons selected** | **Probability of having any video visits in a month   (95% CI)** | **Change in the predicted number of video visits in a month  (95% CI)** |
| --- | --- | --- |
| 0 | 0.59 (0.57, 0.61) | - |
| 1 | 0.63 (0.62, 0.64) | 0.21 (0.7, 0.35) |
| 2 | 0.64 (0.63, 0.65) | 0.58 0.43, 0.74 |
| 3 | 0.64 (0.62, 0.65) | 0.70 (0.53, 0.87) |
| 4 | 0.64 (0.62, 0.66) | 0.66 (0.46, 0.86) |
| 5 | 0.61 (0.58, 0.64) | 0.51 (0.27, 0.74) |
| 6 | 0.61 (0.57, 0.65) | 0.31 (0.01, 0.61) |
| 7 | 0.61 (0.55, 0.66) | 0.36 (-0.06, 0.77) |
| 8 | 0.57 (0.49, 0.66) | -0.51 (-1.04, 0.01) |
| 9 | 0.55 (0.41, 0.68) | -0.59 (-1.39, 0.21) |
| 10+ | 0.67 (0.45, 0.89) | -0.88 (-1.98, 0.21) |
|  |  |  |
| Number of Consult reasons selected (Linear) | 1.00 (0.99, 1.01) | 1.02 (1.01, 1.02) |

Models were adjusted for age at tablet receipt, gender, race, ethnicity and rurality of home
